# Quantitative comparisons of pulmonary artery hemodynamics before and after Pulsta valve implantation in patients with Tetralogy of Fallot using computational fluid dynamics

**DOI:** 10.3389/fcvm.2025.1586134

**Published:** 2025-06-12

**Authors:** Seung Min Baek, Kieun Choi, Sang Yun Lee, Hong Gook Lim, Gi Beom Kim, Jongmin Seo

**Affiliations:** ^1^ Department of Pediatrics, Seoul National University Children’s Hospital, Seoul, Republic of Korea; ^2^ Department of Mechanical Engineering, College of Engineering, Kyung Hee University, Yongin, Gyeonggi-do, Republic of Korea; ^3^ Department of Thoracic and Cardiovascular Surgery, Seoul National University Children’s Hospital, Seoul, Republic of Korea

**Keywords:** computational fluid dynamics, percutaneous pulmonary valve implantation, tetralogy of fallot, cardiac magnetic resonance imaging, pulmonary regurgitation

## Abstract

**Background and objectives:**

The evaluation of percutaneous pulmonary valve implantation (PPVI) performance has been predominantly confined to assessing changes in the right ventricular volume using magnetic resonance imaging (MRI). This study aimed to evaluate the hemodynamic changes in the pulmonary arteries following PPVI using computational fluid dynamics (CFD) in patients with Tetralogy of Fallot.

**Methods:**

We conducted CFD analysis based on MRI scans performed before and after PPVI using Pulsta valve in nine patients who underwent PPVI between 2016 and 2021. Statistical analysis, including Wilcoxon rank-sum tests and multivariable linear regression, was performed to examine the associations between CFD data and non-CFD factors, as well as changes in these parameters after PPVI.

**Results:**

Before PPVI, forward and backward flow velocities in the right pulmonary artery (RPA) were higher than those in the left pulmonary artery (LPA) and main pulmonary artery (MPA) (forward: MPA/RPA/LPA = 19.9/32.7/19.3 cm/s, backward: MPA/RPA/LPA = 10.1/17.0/9.1 cm/s). After PPVI, velocities decreased (forward: MPA/RPA/LPA = 13.3/14.2/8.3 cm/s, backward: MPA/RPA/LPA = 2.3/2.6/1.7 cm/s), reducing the differences among PAs. After PPVI, the vorticity (RPA; 3.9–1.6/s, *p* = 0.008, LPA; 4.4–1.8/s, *p* = 0.011, MPA; 5.4–1.5/s, *p* = 0.008), and energy dissipation (104.1–38.1 mW, *p* = 0.028) decreased significantly, whereas changes in the Womersley and Reynolds numbers were not statistically significant. There was no correlation between the right ventricular end-diastolic volume index and energy dissipation, and the changes in each of them were also unrelated to each other.

**Conclusion:**

A deeper understanding of the hemodynamics of pulmonary arteries using CFD can aid in evaluating the effectiveness of PPVI and refining its indications in patients with Tetralogy of Fallot.

## Introduction

Percutaneous pulmonary valve implantation (PPVI) with a self-expandable valve is safe and effective for reducing right ventricular (RV) volume in patients with Tetralogy of Fallot (TOF) (1–3). Quantitative hemodynamic assessment post-PPVI is important not only for predicting long-term outcomes but also for informing future candidate selection by identifying pre-procedural factors associated with favorable hemodynamic responses. Although evaluating hemodynamic changes in both the RV and pulmonary arteries (PA) is essential to fully understand the therapeutic impact, current clinical practice primarily focuses on RV volume, pressure, and dysfunction due to the limitations of conventional imaging modalities (4–7). In particular, post-PPVI assessment of PA flow is often hindered by metallic stent-induced artifacts in magnetic resonance imaging (MRI).

Recently, computational fluid dynamics (CFD) has emerged as a promising tool to overcome these limitations by enabling patient-specific simulations of cardiovascular flow. CFD allows for detailed hemodynamic analysis in regions not easily accessible through conventional imaging, using three-dimensional (3D) anatomical models derived from imaging data and incorporating boundary conditions from clinical measurements. This technique enables the quantification of important indicators such as flow reversal, vorticity (Vo), and energy dissipation (ED), which are essential for assessing flow efficiency and valve performance. Although previous CFD studies have provided valuable insights into PA morphology, pulmonary regurgitation (PR), and energy efficiency in repaired TOF (8–10), many have relied on generalized or idealized approaches, such as combining geometries from multiple patients or assuming steady-state flow conditions.

In summary, current evaluation methods focus solely on RV assessment due to the technical limitations of MRI, while CFD-based studies that could address these limitations have primarily been conducted using oversimplified or non-patient-specific modeling approaches. To address this gap, this study employed patient-specific CFD in nine patients with TOF who underwent PPVI with a Pulsta transcatheter pulmonary valve (TPV), integrating pre- and post-MRI flow and imaging data for each patient. Using this model, we aimed to: (1) conduct a detailed analysis of hemodynamic changes before and after PPVI, and (2) identify factors that could predict significant hemodynamic improvements following the procedure.

## Materials and methods

Nine patients who underwent PPVI with Pulsta TPV between 2016 and 2021 were selected from the medical records of Seoul National University Children's Hospital. Demographic data and medical history, including surgical history, echocardiography, and pre-PPVI computed tomography (CT), were reviewed. Cardiac catheterization data immediately before the procedure and details of the Pulsta TPV, such as valve diameter, length, and insertion site, were recorded. Insertion sites were classified as proximal, middle, or distal (Supplementary Figure S1). Cardiac MRI scans performed before and after PPVI were reviewed.

On echocardiography, a transpulmonary peak velocity >2 m/s indicated clinically-meaningful PS. Tricuspid regurgitation (TR) was graded by two pediatric cardiologists (S. Y. L. and S. M. B.) through an image review. On cardiac CT, the main pulmonary artery (MPA) shape was classified into five categories as described by Schievano et al*.* (11). Accompanying right ventricular outflow tract (RVOT) aneurysm or branch PA stenosis was noted.

On catheterization, elevated RV systolic pressure (RVSP), RV end-diastolic pressure (RVEDP), and left ventricular end-diastolic pressure (LVEDP) were defined as >30, >6, and >12 mm Hg, respectively. Pulmonary vascular resistance (PVR) was calculated using LVEDP and LV O_2_ saturation instead of mean left atrial pressure and pulmonary venous O_2_ saturation.

On cardiac MRI, pulmonary regurgitation fraction (PR%) was calculated as the ratio of the integral of the backward flow to that of the forward flow across the MPA cross-section. RV ejection fraction (RVEF), RV end-diastolic volume index (RVEDVI), RV end-systolic volume index (RVESVI), RV cardiac index (RVCI), and RV stroke volume index (RVSVI) were recorded.

**Figure 1:**
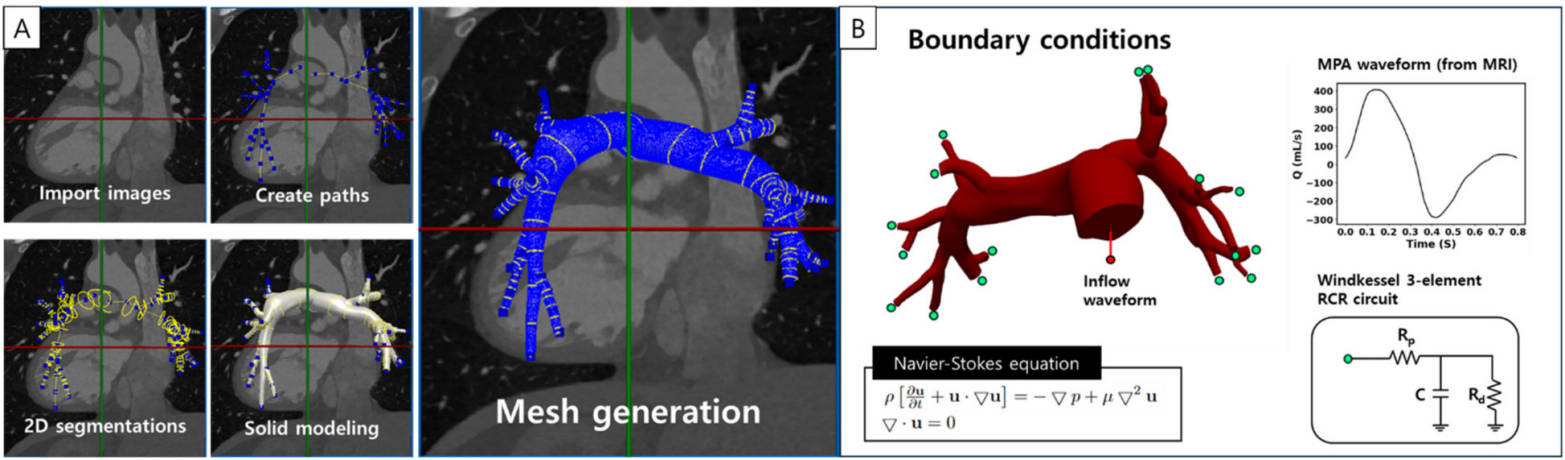
**(A)** Patient-specific three-dimensional pulmonary artery modeling involves path determination, segmentation, solid model, and mesh generation. **(B)** Boundary conditions for the pulmonary artery model. The inflow waveform is obtained from magnetic resonance imaging (MRI) of the main pulmonary artery, and outlet boundary conditions are assigned using the Windkessel resistance-compliance-resistance (RCR) model at each outlet. The Navier-Stokes equation consists of the flow velocity **u**, pressure p, fluid density ρ, and dynamic viscosity μ. MPA, main pulmonary artery.

CFD simulations were conducted by applying the area-averaged flow information from MRI to a patient-specific 3D PA model as boundary conditions. Both pre- and post-operative models were constructed using patient data obtained from MRI and catheter measurements, ensuring that the three-dimensional anatomical models and boundary conditions accurately capture the patient's physiological response. For patient-specific anatomical modeling of the PAs, the DICOM files of cardiac MRI before and after PPVI were loaded into the SimVascular program. One-dimensional paths through the vessel center were created, two-dimensional segmentations were identified by classifying the vessel boundaries, and segmentations were lofted to create 3D anatomic geometry. The model was discretized using millions of tetrahedral element meshes (Figure 1A). Approximately ten branches were reconstructed for both the left pulmonary artery (LPA) and right pulmonary artery (RPA), and a three-element Windkessel model was applied to the outlets of each branch to simulate the physiological response of the downstream vessels. The total PA resistance was calculated using the individual flow rates and mean PA pressure. This resistance was distributed to the LPA and RPA based on the MRI-measured flow distribution. Branch resistances were further distributed based on the branch cap area, applying Murray's law with a modified exponent of 2.3 for PAs $\left(i.e.,\;Q\;\propto r^{2.3}\right)$. Flow splits from the simulation matched the MRI-measured patient-specific flow splits within a 10% error for all patients. A parabolic flow waveform entering the MPA, measured using MRI, was applied at the inlet of the PA model above the RVOT. Since the inlet surface was not circular, velocity vectors were spatially mapped onto the inlet mesh nodes based on their spatial positions. At each time point, the flow rate was converted to a cross-sectional mean velocity, which was then distributed across the inlet surface to preserve the total flow while maintaining a parabolic profile shape.

Cardiovascular simulations were performed using the svSolver program, which solves the incompressible Navier-Stokes and continuity equation (Figure 1B). Blood density was set to 1.06 g/cm^3^ and viscosity to 0.04 g/cm/s. The vessel walls in all PA models were assumed to be rigid. Simulations were run for six cardiac cycles to ensure steady-state flow conditions, and hemodynamic factors were calculated using data from the last cycle to discard the initial data.

The hemodynamic parameters calculated using CFD include the Reynolds number (Re), Womersley number (Wo), vorticity, and energy dissipation. The Womersley number, related to the frequency of pulsatile flow (9), normally ranges from 14 to 21 in the pulmonary artery (12). Vorticity (Vo) represents the degree of rotation and swirl in the fluid flow (10); higher vorticity hypothesizes associated with reduced flow efficiency due to increased recirculation. Energy dissipation (ED) has been used to evaluate flow efficiency in the total cavopulmonary connection (TCPC) of Fontan patients and been suggested to correlate with their exercise capacity or liver fibrosis (13, 14). The Reynolds number, Womersley number, and Vo were measured in both the branch PAs and MPA, while ED was measured in the entire PA bed. Measurement locations in both PAs were at a distance from the PA bifurcation equal to the MPA radius, measured using the centerline (Supplementary Figure S2). We compared these values before and after PPVI. The formulas used to calculate Re, Wo, Vo, and ED are as follows:

Reynoldsnumber(Re)=ρ⋅V⋅Lμ

*ρ*: density of fluid, *V*: velocity of fluid, *L*: diameter of vessel, *μ*: viscosity of fluid.

$$\mathrm{Womersley}\;\mathrm{number}\;(\mathrm{Wo})=r\sqrt{\frac{2\pi }{\nu T}}$$

*r*: radius of the blood vessel, ν: kinematic viscosity, *T*: cardiac cycle

$$\mathrm{Vorticity}\;(\mathrm{Vo})=\;\vec{\nabla }\times \vec{V}$$

$\vec{\nabla }$: directional derivative vector, $\vec{V}$: velocity vector

$$\mathrm{Energy}\;\mathrm{dissipation}\;(\mathrm{ED})=\underset{\mathrm{MPA}}{\sum }\left(P+\frac{1}{2}\rho V^{2}\right)*\;A\;*\;V-\underset{\mathrm{outlet}}{\sum }\left(P+\frac{1}{2}\rho V^{2}\right)\;*\;A\;*\;V$$

P: the averaged pressure in a cardiac cycle, *ρ*: density of fluid, *V*: velocity of fluid, A: cross-sectional area of vessel

Mesh independence tests were conducted by increasing the number of elements from 0.5 to 4 million. We confirmed that Vo in the RPA, LPA, and MPA converged to a value with an error of less than 10% against the finest model (4 million mesh), and the flow rate and pressure in the RPA and LPA converged to a result with an error of less than 2% with a 2 million mesh at an edge size of 0.07 mm. EDs were calculated using the average pressure over six cycles, resulting in a convergence of 2.3% with a 2 million mesh against the 4 million mesh result.

### Statistical analysis

Changes post-PPVI were analyzed using the Wilcoxon rank-sum test. Multivariable linear regression analysis was conducted to explore the associations between the CFD data and RVEDVI, and the absolute reduction and ratio of RVEDVI following PPVI. Additionally, multivariable linear regression was used to investigate the association of Vo and ED with non-CFD factors, including demographic data and measurements from MRI, CT, echocardiography, and other CFD parameters. For independent variables categorized based on specific values, such as PS or elevated RVEDP, both categorical and original continuous variables were analyzed. Variables with *p* < 0.05 in univariate analyses were included in the multivariable model, where all variables remained statistically significant (*p* < 0.05, two-sided test). The analyses were performed using SPSS (version 24.0; IBM Corp., Armonk, NY, USA) and R version 3.0.1 (R Foundation for Statistical Computing, Vienna, Austria).

The study adhered to the Declaration of Helsinki and was approved by the Institutional Review Board of Seoul National University Hospital. The requirement for informed consent from the patients was waived by the board owing to the retrospective design and anonymized nature of the data.

## Results

### Clinical information

Among the nine patients, six (66.7%) were female, and all were Korean. All patients had TOF, except for one with pulmonary atresia with an intact ventricular septum (PA IVS) (Table 1). Median age at corrective surgery was 10.0 (IQR, 4.0–19.6) months, and five patients (55.6%) had prior shunt operation. All eight patients with TOF underwent transannular patch widening during corrective surgery. Median age at PPVI was 23.4 (IQR, 19.9–25.8) years, with details of Pulsta TPV size and location shown in Table 1.

**Table 1:** Clinical information and pre-procedural evaluation.

No	Sex	Dx	Operation history		PPVI (Pulsta) 1nformation			Pre-procedural EchoCG				Pre-procedural CT			Cardiac catheterization			
			Shunt	Age (mo)	Age (yr)	D/L (mm)	Site	PS PV (m/s)	TR grade	TAPSE (mm)	LVEF (%)	RVOT aneurysm	MPA shape	Branch PA	RVSP (RVEDP)	MPA Pr s/d/m	LVEDP	TPG/PVR (WU/m2)
1	F	TOF	central	1.8	20.4	28/38	Mid	1.8	trivial	20.6	77.7		str		NA(6)	24/6/13	10	1/0.24
2	F	PA 4S		2.5	25.5	28/38	Prox	1.3	mild	13.9	58.6		str		17 (6)	16/0/9	7	4/1.47
3	F	TOF		8.6	19.9	28/33	Mid	2	trivial	16.1	70.8		str		27 (10)	24//15	14	1.5/0.52
4	M	TOF		23.0	30.8	32/38	Dist	1.75	trivial	22.9	60.4	o	pyr		25 (8)	18/5/10	9	3/0.7
5	F	TOF	LMBT	21.4	23.4	28/38	Dist	1.6	trivial	18.4	55.4	o	pyr		39 (12)	37/6/20	16	5/2.59
6	F	TOF	RMBT	9.2	19.8	26/38	Dist	2.8	trivial	15.3	63.96	o	pyr	RPA stenosis	29 (6)	20/4/12	7	3.5/1.33
7	F	TOF	LMBT	14.0	26	26/38	Dist	1.9	trivial	23.3	66.9	o	pyr		33 (9)	20/6/12	12	0/0
8	F	TOF		NA	19.2	26/38	Mid	1.6	mild	18.7	58.6		str		33 (13)	28/11/18	12	6/2.26
9	M	TOF	LMBT	10.8	23.6	32/38	Mid	1.8	mild	19.8	64.1		str	LPA stenosis	26 (7)	18/5/10	8	2/0.59

CT, computed tomography; D/L, diameter/length; Dist, distal; Dx, diagnosis; EchoCG, Echocardiography; LMBT, left modified Blalock-Taussig shunt; LPA, left pulmonary artery; LVEDP, left ventricular end diastolic pressure; LVEF, left ventricular ejection fraction; MPA, main pulmonary artery; PA IVS, pulmonary atresia with intact ventricular septum; PS, pulmonary stenosis; PPVI, percutaneous pulmonary valve implantation; Prox, proximal; pyr, pyramid; RMBT, right modified Blalock-Taussig shunt; PV, peak velocity; PVR, pulmonary vascular resistance; RPA, right pulmonary artery; RVOT, right ventricular outflow tract; RVSP, right ventricular systolic pressure; RVEDP, right ventricular end-diastolic pressure; s/d/m, systolic/diastolic/mean; str, straight; TAPSE, tricuspid annular plane systolic excursion; TOF, Tetralogy of Fallot; TPG, transpulmonary gradient; TR, tricuspid regurgitation; WU, wood unit.

### Pre-procedural evaluation

On pre-PPVI echocardiography, one patient (Patient 6) had significant PS with a peak velocity of 2.8 m/s. Three patients had mild TR and normal left ventricular ejection fraction. On CT, five patients had a straight-shaped MPA, whereas others had a pyramidal shape. One patient had mild LPA stenosis, and another had moderate mid-RPA stenosis. RVOT aneurysm was observed in four patients.

RVSP and RVEDP were elevated in three and six patients (33.3% and 66.6%, respectively). LVEDP was elevated in two patients (22.2%). One patient had borderline pulmonary hypertension (mean MPA pressure, 20 mmHg), and none of the patients had increased PVR.

### Cardiac MRI before and after PPVI with Pulsta valve

Pre-procedure and post-procedure MRI were conducted at a median of 120 (IQR, 61–260) days and 197 (IQR, 171.5–375) days before and after PPVI, respectively (Supplementary Table S1). After PPVI, RVEDVI, RVCI, RVSVI, and PR% significantly decreased; RVEDVI from 167.4 to 126.9 ml/m^2^ (*p* = 0.008), RVCI from 5.5 to 4.0 L/min/m^2^ (*p* = 0.015), RVSVI from 83.5 to 61.9 ml/m^2^ (*p* = 0.008), and PR% from 44.2% to 14.5% (*p* = 0.008).

### CFD analysis

**Figure 2:**
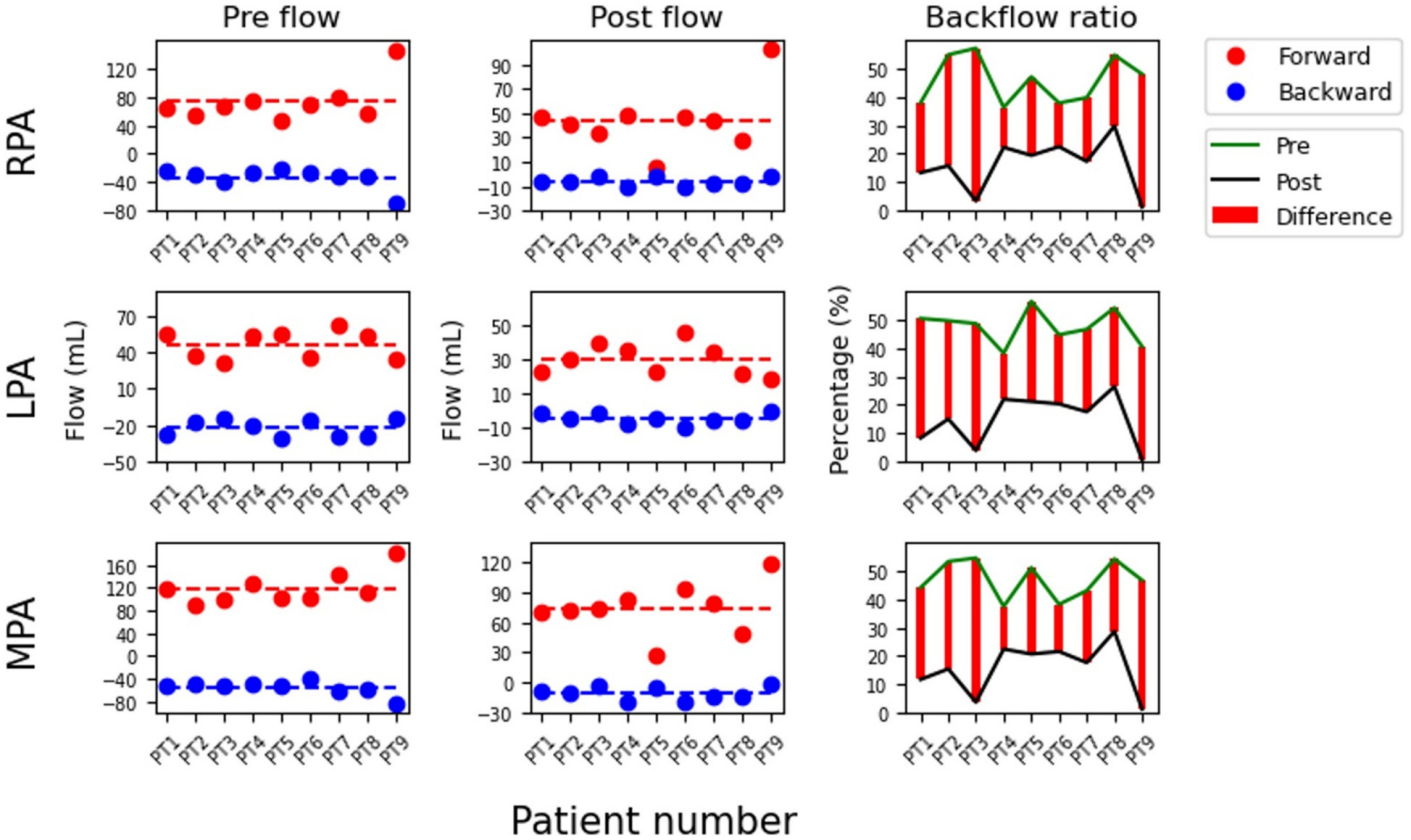
Comparison of forward flow (Forward), backward flow (Backward), and forward-to-backward ratio (Backflow ratio) before and after Pulsta valve implantation in each patient. The values are calculated from the right (RPA), left (LPA), and main pulmonary artery (MPA). (Left) flow represents pre-implantation, (Center) flow represents post-implantation, and (Right) the difference in backflow ratio between pre- and post-implantation. The labels PT1-9 on the *x*-axis denote patient numbers.

Video 1 shows the pre- and post-PPVI pulmonary blood flows in patient 1. Figure 2 shows the integrated forward and backward flow amounts at the MPA, LPA, and RPA for the nine patients. While both flows decreased, backward flow significantly reduced, leading to an overall PR% reduction (RPA; 46.1%–16.1%, *p* = 0.008, LPA; 47.8%–15.1%, *p* = 0.008, MPA; 46.9%–15.8%, *p* = 0.008, Supplementary Table S2).

**Figure 3:**
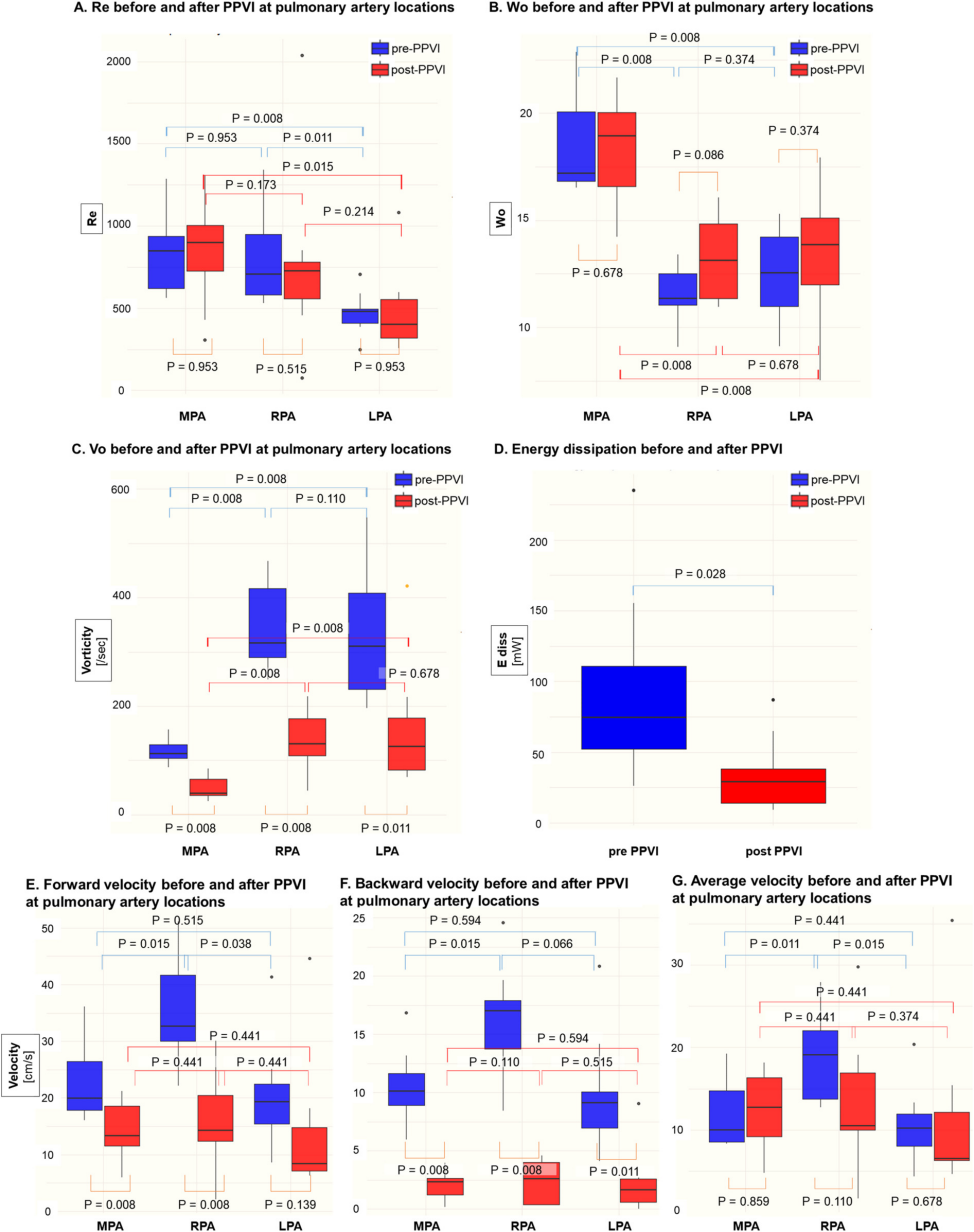
Comparison of computational fluid dynamic indices in the main pulmonary artery (MPA), right pulmonary artery (RPA), and left pulmonary artery (LPA) before and after Pulsta valve implantation. **(A)** Reynolds number (Re), **(B)** Womersley number (Wo), **(C)** vorticity (Vo), **(D)** energy dissipation (E diss), **(E)** forward velocity, **(F)** backward velocity, and **(G)** average velocity were compared at each pulmonary artery segment. PPVI, percutaneous pulmonary valve implantation; Re, Reynolds number; Wo, Womersley number; Vo, vorticity.

Table 2 and Figure 3 summarize the hemodynamic indices before and after PPVI. Vo was lower in the MPA than in the RPA and LPA (*p* = 0.008 for both pre- and post-PPVI), whereas Wo was higher in the MPA (*p* = 0.008 for both pre- and post-PPVI). Re and Wo did not show significant changes post-procedure across all PAs, although Vo significantly decreased at all PA locations (RPA; 3.9–1.6/s, *p* = 0.008, LPA; 4.4–1.8/s, *p* = 0.011, MPA; 5.4–1.5/s, *p* = 0.008), and ED also notably reduced (104.1–38.1 mW, *p* = 0.028). Before PPVI, both forward and backward velocities in the RPA were faster than those in the other PAs (forward: RPA 32.7 vs. MPA 19.9 [*p* = 0.015], LPA 19.3 [*p* = 0.038] cm/s; backward: RPA 17.0 vs. MPA 10.1 [*p* = 0.015], LPA 9.1 [*p* = 0.066] cm/s). These differences diminished after PPVI (forward: RPA 14.2 vs. MPA 13.3 [*p* = 0.441], LPA 8.3 [*p* = 0.441] cm/s; backward: RPA 2.6 vs. MPA 2.3 [*p* = 0.110], LPA 1.7 [*p* = 0.515] cm/s).

**Table 2:** Computational fluid dynamics-derived hemodynamic indices before and after Pulsta valve implantation.

No	MPA						RPA						LPA						ED [mW]	
	Re		Wo		Vo (/s)		Re		Wo		Vo		Re		Wo		Vo (/s)			
	Pre	Post	Pre	Post	Pre	Post	Pre	Post	Pre	Post	Pre	Post	Pre	Post	Pre	Post	Pre	Post	Pre	Post
1	936.0	1,004.2	16.7	14.2	129.2	75.0	698.0	725.6	13.4	11.9	248.7	188.5	704.8	352.6	9.1	13.9	462.2	125.6	55.3	64.7
2	560.9	728.8	18.5	20.3	103.7	35.2	528.8	724.8	11.4	14.8	337.2	90.4	410.1	598.3	11.0	10.5	310.3	178.3	27.7	32.5
3	579.5	899.4	17.2	20.0	87.2	43.7	579.7	557.1	11.0	15.0	295.4	109.2	249.1	544.9	14.2	17.9	195.8	122.0	26.2	21.4
4	849.6	726.7	22.8	21.7	95.4	39.3	907.5	568.5	12.8	16.1	292.3	148.1	590.4	400.3	13.3	16.9	231.4	139.9	110.7	14.1
5	618.7	306.6	20.6	17.6	105.8	24.8	706.4	76.5	9.1	13.3	465.5	43.9	481.3	300.6	12.5	14.4	408.3	78.7	106.8	9.5
6	869.2	977.8	17.1	16.6	113.1	65.6	1,035.4	850.8	9.8	11.2	709.2	218.0	384.8	1,082.1	12.0	7.5	547.2	422.0	234.9	87.0
7	1,077.5	1,006.5	16.8	15.4	129.3	85.4	949.1	782.5	11.4	11.0	283.4	176.9	489.1	554.4	15.3	12.2	208.0	217.2	52.2	38.3
8	649.9	429.0	20.1	20.0	129.6	39.1	580.6	456.0	11.4	11.4	466.9	130.7	418.3	256.9	15.0	15.1	337.1	83.1	155.5	9.4
9	1,285.9	1,307.3	16.5	18.9	157.1	35.7	1,340.8	2,037.6	12.5	13.1	401.2	122.7	495.5	318.1	9.2	12.0	286.0	69.4	74.7	29.2
Mean	825.2	820.7	18.5	18.3	116.7	49.3	814.0	753.3	11.4	13.1	388.9	136.5	469.3	489.8	12.4	13.4	331.8	159.6	93.8	34.0
SD	248.6	310.4	2.23	2.49	21.6	20.8	266.8	533.9	1.37	1.87	144.2	53.4	129.0	254.0	2.31	3.22	120.3	109.6	67.7	26.4
*p*	0.953		0.678		0.008		0.515		0.086		0.008		0.953		0.374		0.011		0.028	

ED, Energy dissipation; LPA, left pulmonary artery; MPA, main pulmonary artery; Re, Reynolds number; RPA, right pulmonary artery; SD, standard deviation; Vo, Vorticity; Wo, Womersley number.

### Correlations between RVEDVI and CFD measurements

**Figure 4:**
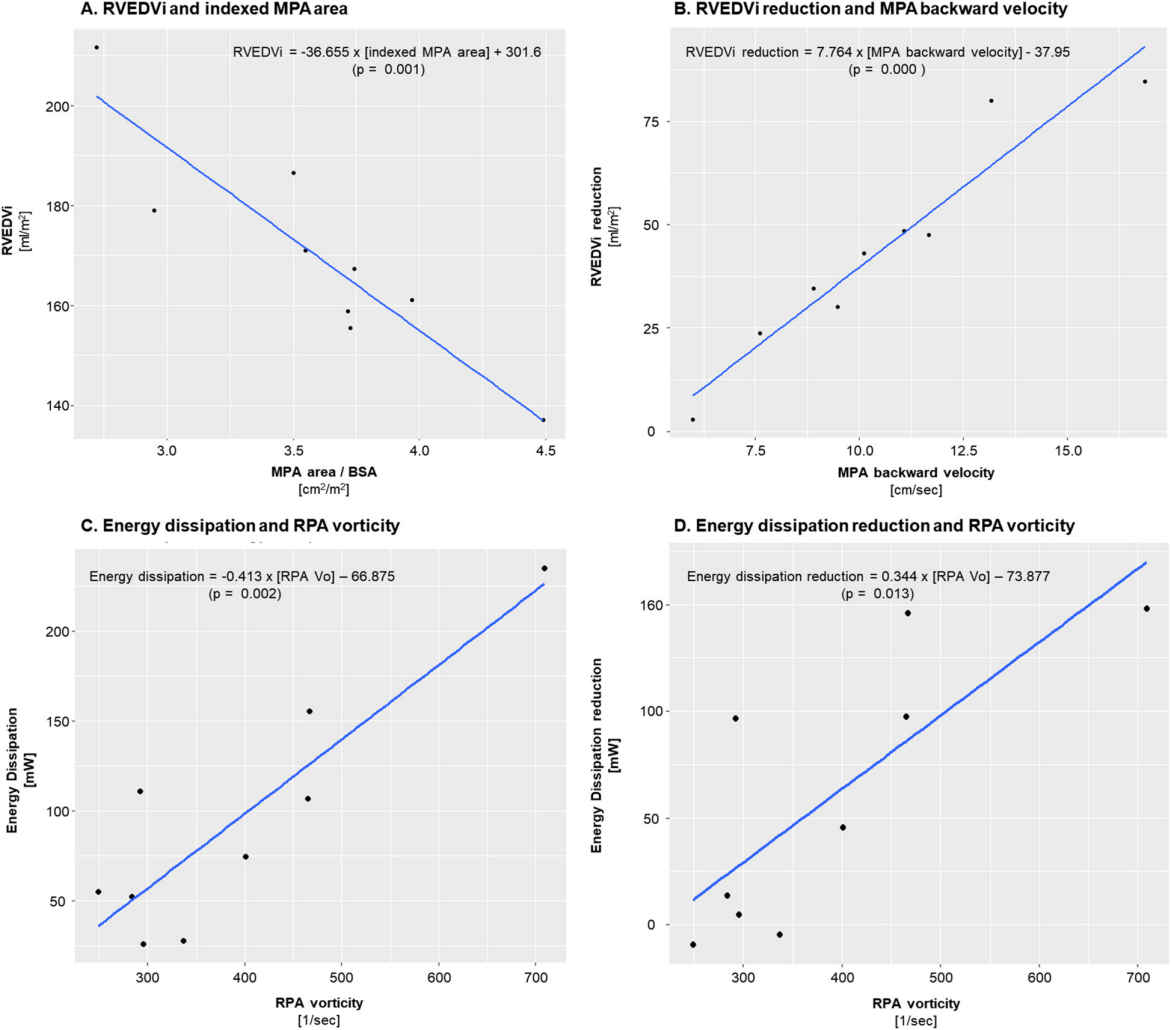
Interrelationships between right ventricular end diastolic volume index (RVEDVi), energy dissipation, and their respective post-procedural changes with relevant measurements. **(A)** Negative correlation between indexed MPA area and pre-procedural RVEDVi. **(B)** Positive correlation between MPA backward velocity and RVEDVi reduction after PPVI. **(C)** Positive correlation between RPA vorticity and total energy dissipation. **(D)** Positive correlation between RPA vorticity and energy dissipation reduction after PPVI. MPA, main pulmonary artery; RPA, right pulmonary artery; BSA, body surface area; PPVI, percutaneous pulmonary valve implantation; Vo, vorticity.

The correlations between the CFD data and MRI RVEDVI, and changes after PPVI are summarized in Table 3. In univariable analysis, MRI RVEDVI correlated with various CFD data, but only MPA Vo and indexed MPA area (MPA area/BSA) were significant (Coefficient B = 0.419 ± 0.168, *p* = 0.047; Coefficient B = −24.41 ± 6.932, *p* = 0.012, respectively) in multivariable analysis. Similarly, RVEDVI reduction and its ratio correlated with various CFD data, with MPA backward velocity being significant in multivariable analysis (Coefficient B = 7.764 ± 0.868, *p* < 0.001 for RVEDVI reduction and Coefficient B = 0.036 ± 0.016, *p* = 0.001 for reduction ratio). Figures 4A,B show the relationship between the significant CFD data and RVEDVI, and its reduction. RVEDVI negatively correlated with MPA-indexed area, whereas RVEDVI reduction positively correlated with MPA backward velocity.

**Table 3:** Correlations of computational fluid dynamic data with right ventricular end diastolic volume index and the change in it after Pulsta valve implantation.

CFD parameters	With RVEDVI				With RVEDVI reduction				With RVEDVI reduction ratio			
	Univariable		Multivariable		Univariable		Multivariable		Univariable		Multivariable	
	Coefficient B	*P*	Coefficient B	*P*	Coefficient B	*P*	Coefficient B	*P*	Coefficient B	*P*	Coefficient B	P
RPA forward Q	0.5 ± 0.48	**0** **.** **043**			0.53 ± 0.64	0.090			0.002 ± 0.004	0.235		
LPA forward Q	−0.29 ± 1.59	0.680			0.19 ± 1.96	0.823			0.002 ± 0.01	0.728		
MPA forward Q	0.48 ± 0.52	0.067			0.61 ± 0.62	0.054			0.002 ± 0.004	0.152		
RPA forward velocity	0.9 ± 1.92	0.303			0.95 ± 2.41	0.383			0.004 ± 0.012	0.509		
LPA forward velocity	0.86 ± 1.92	0.324			0.09 ± 2.53	0.932			−0.001 ± 0.013	0.897		
MPA forward velocity	2.74 ± 1.2	**0** **.** **001**			3.35 ± 1.48	**0** **.** **001**			0.015 ± 0.01	**0** **.** **01**		
RPA backward Q	0.98 ± 0.97	**0** **.** **049**			1.15 ± 1.22	0.061			0.005 ± 0.007	0.145		
LPA backward Q	−0.41 ± 2.72	0.731			0.44 ± 3.33	0.763			0.004 ± 0.016	0.583		
MPA backward Q	1.16 ± 1.08	**0** **.** **038**			1.68 ± 1.05	**0** **.** **007**			0.008 ± 0.006	**0** **.** **023**		
RPA backward velocity	1.62 ± 3.69	0.333			2.25 ± 4.4	0.267			0.011 ± 0.022	0.301		
LPA backward velocity	1.28 ± 3.64	0.433			0.14 ± 4.68	0.948			0 ± 0.023	0.973		
MPA backward velocity	5.88 ± 2.72	**0** **.** **001**			7.76 ± 2.05	**0** **.** **000**	7.76 ± 2.05	**0.000**	0.036 ± 0.016	**0** **.** **001**	0.036 ± 0.016	**0.001**
RPA Re	0.04 ± 0.06	0.142			0.04 ± 0.08	0.296			0 ± 0	0.537		
RPA Wo	4.38 ± 13.19	0.458			3.05 ± 16.62	0.677			0.005 ± 0.084	0.888		
RPA Vo	0 ± 0.13	0.945			−0.04 ± 0.16	0.611			0 ± 0.001	0.612		
LPA Re	0.04 ± 0.14	0.566			0 ± 0.18	0.998			0 ± 0.001	0.787		
LPA Wo	−5.69 ± 6.41	0.074			−0.9 ± 9.98	0.838			0.005 ± 0.05	0.837		
LPA Vo	0.03 ± 0.16	0.712			−0.06 ± 0.19	0.506			0 ± 0.001	0.462		
MPA Re	0.06 ± 0.05	**0** **.** **036**			0.06 ± 0.07	0.082			0 ± 0	0.209		
MPA Wo	−7.2 ± 5.56	**0** **.** **018**			−7.8 ± 7.71	**0** **.** **048**			−0.038 ± 0.04	0.059		
MPA Vo	0.84 ± 0.45	**0** **.** **003**	0.419 ± 0.41	**0.047**	0.92 ± 0.68	**0** **.** **015**			0.004 ± 0.004	**0** **.** **049**		
Energy Dissipation	−0.07 ± 0.27	0.571			−0.13 ± 0.32	0.363			−0.001 ± 0.002	0.356		
RPA area/BSA	21.4 ± 42.56	0.272			17.8 ± 54.86	0.467			0.07 ± 0.279	0.574		
LPA area/BSA	−13.4 ± 26.25	0.268			4.63 ± 35	0.764			0.056 ± 0.17	0.46		
MPA area/BSA	−36.7 ± 15.35	**0** **.** **001**	−24.41 ± 16.96	**0.012**	−44.7 ± 18.96	**0** **.** **001**			−0.209 ± 0.12	**0** **.** **004**		

Bold values indicate statistically significant *p*-values (*p* < 0.05).

BSA, body surface area; LPA, left pulmonary artery; MPA, main pulmonary artery; PR%, pulmonary regurgitation fraction; Q, blood flow rate; Re, Reynolds number; RPA, right pulmonary artery; RVEDVI, right ventricular end-diastolic volume index; Vo, vorticity; Wo, Womersley number.

### Correlation of Vo and ED with non-CFD factors and other CFD data

MPA Vo exhibited significant correlations with RVEDVI and RVSVI in univariable analysis (Supplementary Table S3). In multivariable analysis, only MPA forward velocity showed significant correlation (Coefficient B = 2.749 ± 1.09, *p* = 0.003). Conversely, RPA and LPA Vo showed no significant association with non-CFD factors, aside from a weak correlation between RVEDP and LPA Vo in univariable analysis. RPA and LPA Vo showed significant correlation only with ED in multivariable analysis (Coefficient B = 1.871 ± 0.751, *p* = 0.002; Coefficient B = 0.995 ± 0.849, *p* = 0.03, respectively).

ED showed no significant correlation with non-CFD factors, except for a weak correlation with PS peak velocity on echocardiography (*p* = 0.07; Table 4). ED correlated only with RPA Vo in univariable analysis (Coefficient B = 0.413 ± 0.2, *p* = 0.002). ED reduction correlated with transpulmonary pressure gradient and RPA Vo in univariable analysis, but only RPA Vo remained significant in multivariable analysis (Coefficient B = 0.34 ± 0.20, *p* = 0.013). The ED reduction ratio correlated with age at corrective surgery in both univariable and multivariable analyses. Figures 4C,D show the positive correlation between RPA Vo and ED, and between RPA Vo and ED reduction.

**Table 4:** Correlations of energy dissipation with non-CFD factors and other CFD measurements.

Factors and measurements		With energy dissipation				With energy dissipation reduction				With energy dissipation reduction Ratio			
		Univariable		Multivariable		Univariable		Multivariable		Univariable		Multivariable	
		Coefficient B	*P*	Coefficient B	*P*	Coefficient B	*P*	Coefficient B	*P*	Coefficient B	*P*	Coefficient B	*P*
Clinical information	Shunt op	24.73 ± 93.43	0.62			−1.66 ± 89.27	0.972			−0.007 ± 0.622	0.982		
	Age at op	2.06 ± 6.65	0.562			3.81 ± 5.74	0.235			0.045 ± 0.03	**0** **.** **021**	0.045 ± 0.03	0.021
PPVI information	Age at PPVI	−4.61 ± 12.65	0.498			−2.26 ± 12.17	0.727			0.013 ± 0.085	0.782		
	Pulsta site	48.9 ± 61.02	0.16			45.29 ± 57.47	0.166			0.373 ± 0.372	0.0895		
EchoCG	PS peak velocity	102.8 ± 94.42	0.07			57.14 ± 105.58	0.324			0.151 ± 0.784	0.717		
	TR grade	−20.25 ± 94.02	0.686			−11.2 ± 88.89	0.812			−0.082 ± 0.619	0.802		
	TAPSE	−2.42 ± 15.29	0.766			−0.73 ± 14.42	0.924			0.033 ± 0.097	0.522		
	LVEF	−3.26 ± 6.79	0.378			−5.25 ± 5.53	0.105			−0.04 ± 0.036	0.0667		
CT	MPA shape	58.28 ± 84.86	0.22			52.47 ± 80.37	0.241			0.392 ± 0.55	0.205		
	RVOT aneurysm	22.98 ± 98.9	0.663			12.02 ± 93.69	0.809			0.161 ± 0.644	0.639		
	Branch PAS	78.464 ± 97.83	0.16			47.5 ± 100.74	0.386			0.215 ± 0.726	0.580		
Cath	RVSP	3.76 ± 8.05	0.395			−0.001 ± 0.001	0.275			0.039 ± 0.037	0.0856		
	RVEDP	2.31 ± 18.85	0.817			9.46 ± 16.31	0.293			0.098 ± 0.1	0.0944		
	LVEDP	−4.44 ± 15.5	0.592			0.09 ± 14.86	0.991			0.036 ± 0.1	0.503		
	TPG	17.71 ± 22.15	0.161			22.59 ± 17.4	**0** **.** **038**			0.128 ± 0.139	0.113		
MRI	RVEF	1.81 ± 8.85	0.701			0.26 ± 8.39	0.953			−0.025 ± 0.056	0.411		
	RVEDVI	−0.7 ± 2.31	0.571			−1.13 ± 2.06	0.318			−0.007 ± 0.015	0.362		
	RVESVI	−0.99 ± 3.04	0.541			−1.05 ± 2.82	0.488			−0.001 ± 0.02	0.93		
	PR%	0.63 ± 8.36	0.886			2.78 ± 7.57	0.496			0.018 ± 0.053	0.535		
CFD	MPA Re	0.01 ± 0.2	0.945			−0.03 ± 0.19	0.786			0 ± 0.001	0.942		
	MPA Wo	6.67 ± 21.98	0.571			13.95 ± 18.43	0.182			0.121 ± 0.117	0.0821		
	MPA Vo	0.23 ± 2.31	0.849			0.01 ± 2.18	0.990			0.001 ± 0.015	0.894		
	RPA Re	0.07 ± 0.18	0.465			0.04 ± 0.17	0.644			0.001 ± 0.001	0.428		
	RPA Wo	−19.56 ± 33.5	0.29			−19.9 ± 30.89	0.247			−0.111 ± 0.224	0.364		
	RPA Vo	0.41 ± 0.17	**0** **.** **002**	0.41 ± 0.17	0.002	0.34 ± 0.2	**0** **.** **013**	0.34 ± 0.2	**0.013**	0.002 ± 0.002	0.169		
	LPA Re	−0.03 ± 0.39	0.87			−0.06 ± 0.36	0.736			0 ± 0.003	0.848		
	LPA Wo	3.67 ± 21.51	0.748			8.42 ± 19.35	0.422			0.071 ± 0.131	0.322		
	LPA Vo	0.36 ± 0.32	0.064			0.21 ± 0.36	0.279			0 ± 0.003	0.914		
	MPA area/BSA	46.71 ± 89.45	0.34			58.22 ± 78.92	0.191			0.309 ± 0.583	0.333		
	RPA area/BSA	−74.09 ± 110.74	0.231			−96.16 ± 91.42	0.078			−0.703 ± 0.616	0.0604		
	LPA area/BSA	−1.27 ± 76.55	0.975			5.95 ± 71.65	0.875			0.051 ± 0.498	0.846		
	RPA vel/MPA vel	70.82 ± 89.70	0.166			84.15 ± 74.90	0.064			0.612 ± 0.505	**0** **.** **0493**		
	LPA vel/MPA vel	−24.74 ± 141.62	0.742			−14.65 ± 133.47	0.836			−0.134 ± 0.927	0.785		

Bold values indicate statistically significant *p*-values (*p* < 0.05).

BSA, body surface area; BPA, branch pulmonary artery; EchoCG, Echocardiography; LPA, left pulmonary artery; LVEDP, left ventricular end diastolic pressure; LVEF, left ventricular ejection fraction; MPA, main pulmonary artery; op, operation; PPVI, percutaneous pulmonary valve implantation; PR%, pulmonary regurgitation fraction; PS, pulmonary stenosis; TAPSE, tricuspid annular plane systolic excursion; TR, tricuspid regurgitation; Q, blood flow rate; Re, Reynolds number; RPA, right pulmonary artery; RVEDP, right ventricular end diastolic pressure; RVEDVI, right ventricular end diastolic volume index; RVEF, right ventricular ejection fraction; RVESVI, right ventricular end systolic volume index; RVOT, right ventricular outflow tract; RVSP, right ventricular systolic pressure; vel, velocity; Vo, vorticity; Wo, Womersley number.

## Discussion

This study investigated hemodynamic changes following PPVI using CFD. Post-procedure, there was no significant decrease in Re or Wo, potentially because pre-procedurally, Re was not notably high and Wo was within the normal range (14–21) in healthy PAs (12). Conversely, Vo and ED decreased significantly, indicating hemodynamic improvement following PPVI. The higher Wo and lower Vo observed in the MPA compared to the branch PAs can be attributed to differences in the vascular radius, which persisted after the intervention.

Contrary to previous studies using phase contrast MRI, which showed higher velocity, Vo, PR%, and cross-sectional area in the LPA compared to RPA (15–17), our data revealed higher velocity and Vo in the RPA, with no significant difference in the cross-sectional area or PR% between the two (RPA vs. LPA area = 2.13 cm^2^ vs. 2.53 cm^2^, *p* = 0.374; RPA vs. LPA PR% = 46.1% vs. 47.8%, *p* = 0.52, Supplementary Table S2). Siripornpitak et al. found that approximately 24% patients had a higher PR% in the RPA, indicating that the LPA does not always exhibit a higher velocity/Vo or PR% (16). Factors such as length and branching angle of the PA and direction and morphology of the MPA likely influence which side shows higher velocity/Vo. A larger cohort may yield results similar to those of previous studies.

However, an imbalanced blood flow between the branch PAs favoring the RPA or LPA causes increased velocity and Vo in the PA receiving the greater flow, and increased Vo contributes to a higher ED (Table 4), reflecting the hemodynamic disadvantage associated with PR. These results align with the assumption mentioned earlier that vorticity may have a negative correlation with energy efficiency. Furthermore, blood flow imbalance pre-PPVI was resolved post-PPVI (Pre: RPA vs. LPA = 73.5 ml/s vs. 46.2 ml/s, *p* = 0.015, post: RPA vs. LPA = 44.2 ml/s vs. 30.2 ml/s, *p* = 0.139, Figure 2 and Supplementary Table S2), indicating hemodynamic improvement following PPVI. Therefore, considering the increase in Vo in branch PAs can help refine the indications for PPVI.

The negative correlation between indexed MPA area and RVEDVI was unexpected. In multivariable analysis, RVESVI also significantly negatively correlated with the indexed MPA area (Coefficient B = −25.82 ± 12.29, *p* = 0.004). We expected a positive correlation between the MPA area and RVEDVI if the RV stroke volume increased within the same subject, as both the MPA and RV would likely enlarge together. However, in different subjects with similar RV stroke volume increases, the varying compliance of the MPA and RV may influence which side expands more. Thus, a less compliant MPA may cause volumetric overload, primarily affecting the RV rather than the MPA.

RVEDVI reduction and its ratio were significantly positively correlated with the MPA backward velocity. Given the same PR volume, a higher MPA backward velocity is expected when the MPA area is narrow or the RV is compliant. Thus, principles similar to those discussed regarding volume overload differences between the RV and MPA due to compliance variations could apply to the relationship between RVEDVI reduction/ratio and MPA backward velocity.

The ED reduction ratio showed a mildly positive correlation with age at the initial corrective surgery. High-speed jet flow biases the flow distribution to branch PAs based on the arterial direction and angle, potentially affecting LPA and RPA development with delayed corrective operations. However, age at corrective operation exhibited no correlation with ED or ED reduction but only with the ED reduction ratio. Relative velocity in the RPA compared to the MPA and indexed RPA area weakly and consistently correlated with ED reduction and ratio. Collinearity between relative velocity in RPA and age at corrective operation (Coefficient B = 10.242 ± 8.352, *p* = 0.047) suggested that a higher RPA velocity and vorticity were associated with overall ED and changes post-PPVI.

Current indications for pulmonary valve replacement rely primarily on RV volume indices obtained from MRI when the residual pulmonary valve pathology is PR (4–7). However, the impact of PR on the entire circulatory system may extend beyond RV volume overload to include the efficiency of blood flow within the PAs. Since no clear correlation was observed between RVEDVI or RVESVI and CFD indices, and the factors associated with the reduction in RVEDVI and ED were distinct, RV volume overload and PA hemodynamics may be independent of each other. Therefore, future research aimed at establishing CFD numerical criteria to predict significant hemodynamic improvements could refine the indications for PPVI.

Loke et al. recently demonstrated that CFD can assess intracardiac flow patterns in the right ventricle, revealing significant changes in Vo and ED after pulmonary valve replacement (18). Their study emphasized that conventional MRI-derived volume metrics alone may not fully capture the hemodynamic impact of pulmonary regurgitation or its correction. Combined with our findings in the pulmonary arteries, this highlights the utility of CFD as a comprehensive tool for post-interventional evaluation. To refine indications for PPVI, future studies should assess both intracardiac and pulmonary arterial flow dynamics.

Furthermore, PPVI indications in patients with combined PS and PR remain debatable (3). While our study primarily focused on PR-dominant lesions, the hemodynamic impact of PS remains an important yet underexplored factor in CFD-based assessments. A recent virtual surgery study using patient-specific models demonstrated that variations in branch PA angulation after surgical arterioplasty influenced postoperative flow patterns, wall shear stress, and ED in TOF patients with PS (19). This highlights the potential of CFD not only in evaluating PR-related hemodynamics, but also in capturing the complex flow disturbances caused by PS. Incorporating such insights may help refine PPVI indications in patients with mixed lesions of PS and PR. Our study suggested a potential association between ED and peak velocity (*p* = 0.07; Table 4), though the limited sample size precludes definitive conclusions. Specifically, in Patient 6, who exhibited the only significant PS in our cohort, pronounced pre-PPVI ED was observed, which markedly decreased post-procedure. Therefore, evaluating ED in patients undergoing PPVI for pulmonary steno-insufficiency could enhance the assessment of PPVI effectiveness. While this study primarily focused on patients undergoing Pulsta TPV implantation because of PR, including more patients with concurrent PS could provide further clarity regarding these relationships.

Among the hemodynamic factors, factors other than Wo exhibited an average variability of 46% compared with the mean, with ED showing a variability of over 70%. This variability underscores the importance of a patient-specific clinical assessment. CFD enables the visualization of individual blood flow in areas where standard measurements are limited, offering a more accurate calculation of hemodynamic factors through 3D flow field computation. This noninvasive investigation can significantly contribute to preprocedural treatment planning decisions and prediction of treatment outcomes.

## Limitations

This study included a small number of patients, which may have limited the statistical analysis. Due to the limited number of patients with significant PS or branch PA stenosis, statistical analysis of the influence of these factors was constrained. Furthermore, this study lacks conclusive evidence on whether improvements in ED or Vo can effectively alleviate patient symptoms or enhance prognosis. Resolving RV volume overload and decreasing ED/Vo within the PAs may collectively impact patient prognosis; however, further research is needed to substantiate this hypothesis.

CFD has certain limitations. First, we used time-series in-plane averaged flow rate data from MRI measurements above the Pulsta valve to set the inlet conditions. Due to the lack of detailed measurements of the in-plane velocity distributions, we applied a Poiseuille flow-type velocity distribution. This simplified velocity condition may lead to errors compared with a realistic velocity distribution, as the valve motion may create a more irregular in-plane velocity distribution.

Additionally, we performed simulations assuming that the PA wall is rigid while neglecting vessel wall compliance. Reflecting on the large PA deformation during systole and diastole in the simulation could provide physiologically appropriate results. Indeed, previous studies have shown that vessel wall deformation significantly affects hemodynamic parameters, including energy dissipation (20). However, modeling and simulating large-vessel wall deformations using CFD is computationally challenging. Recent CFD studies employed fluid-structure-interaction (FSI) through the Arbitrary Lagrangian-Eulerian (ALE) framework for simulating vessel wall deformation (21, 22); however, their applications are limited. Future studies should investigate the hemodynamics using an ALE-FSI CFD solver.

In conclusion, understanding the PA hemodynamics using CFD can enhance the evaluation of the effectiveness of PPVI and refine its indications.

## Data Availability

The original contributions presented in the study are included in the article/Supplementary Material, further inquiries can be directed to the corresponding authors.
