# Supplementary material for: Quantitative comparisons of pulmonary artery hemodynamics before and after Pulsta valve implantation in patients with Tetralogy of Fallot using computational fluid dynamics
Source: Front Cardiovasc Med. 2025 Jun 12;12:1586134. doi: 10.3389/fcvm.2025.1586134 (PMC12198252; doi:10.3389/fcvm.2025.1586134)
Supplement: Supplementary file 1 [file Datasheet1.pdf]

Supplemental Materials

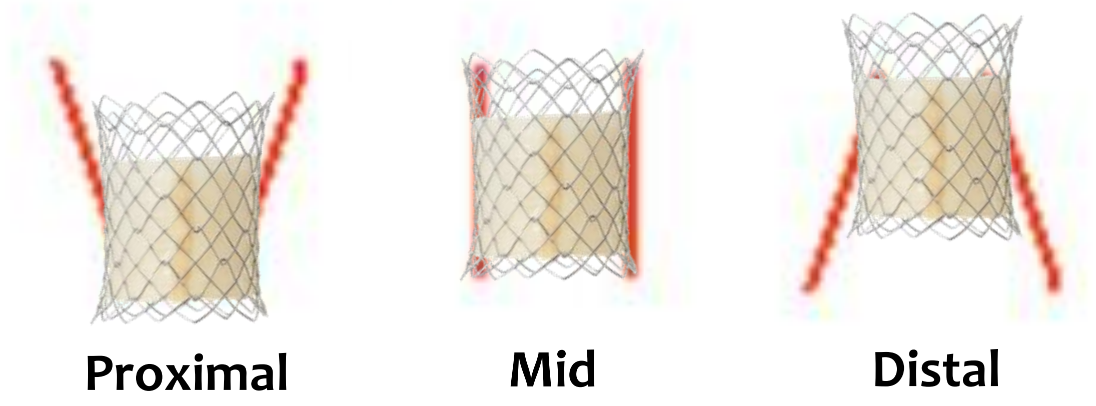

Fig. S1. The insertion site of Pulsta TPV was based on the shape of the main pulmonary artery. A Pulsta valve with a total length of 38 mm can be inserted at the proximal, mid, or distal parts of the main pulmonary artery, depending on the main pulmonary artery morphology.

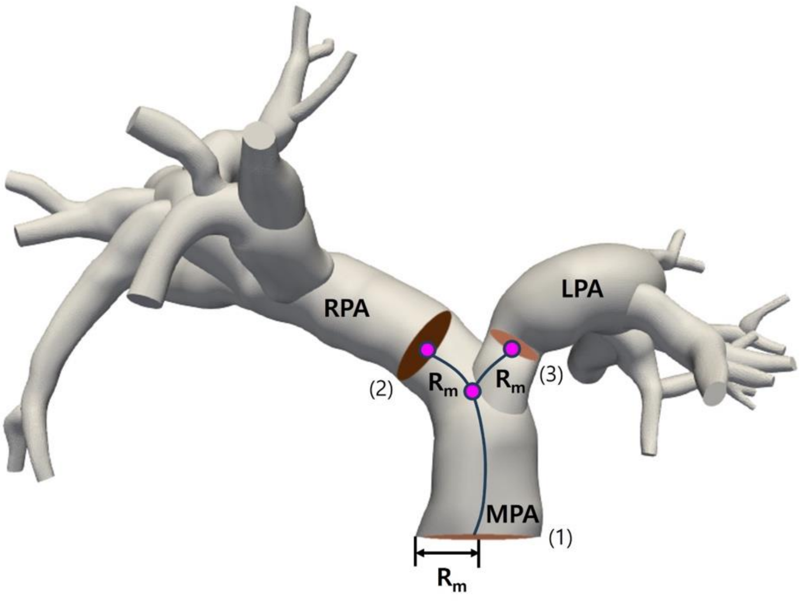

Fig. S2. Hemodynamic indices were calculated in slice (1) in the main pulmonary artery (MPA) and slices (2) and (3) in the right pulmonary artery (RPA) and left pulmonary artery (LPA), respectively. Slices (2)–(3) are located at a distance of the MPA radius (Rm) from the bifurcation point of the MPA (center dot).

Table S1. Cardiac magnetic resonance imaging data before and after Pulsta valve implantation

**No**	**Sex**	**Dx**	**Time interval** (days)		**BSA** (m2)		**RVEF** (%)		**RVEDVI** (mL/m2)		**RVESVI** (mL/m2)		**RVCI** (L/min/m2)		**RVSVI** (mL/m2)		**PR%** (%)	
			pre	post	pre	pre	post	post	pre	post	pre	post	pre	post	pre	post	pre	post
1	F	TOF	44	176	1.28	1.31	51.9	49.5	186.5	139.0	89.7	70.1	6.2	3.8	91.8	62.1	38.4	7.0
2	F	PA IVS	1	197	1.44	1.47	50.9	47.2	171.0	140.9	84	74.4	6.4	4.6	87.0	66.5	53.2	14.4
3	F	TOF	363	365	1.42	1.40	41.3	42.6	155.5	112.4	91.3	64.5	3.8	4.4	64.1	47.9	44.2	4.1
4	M	TOF	157	385	1.79	1.80	48.5	52.7	137.1	134.2	70.9	72.2	5.5	4.5	66.8	61.9	39.9	23.2
5	F	TOF	115	154	1.59	1.56	36.8	44.1	158.9	124.3	100.5	69.5	6.0	4.0	77.0	55.7	49.5	19.0
6	F	TOF	149	210	1.36	1.38	48.0	46.7	167.4	143.7	87	76.6	5.3	3.8	80.4	67.1	43.0	19.2
7	F	TOF	78	181	1.58	1.59	46.6	42.9	179.0	99.0	95.6	56.6	5.0	2.7	83.5	39.8	42.5	14.5
8	F	TOF	120	167	1.36	1.33	52.4	52.7	161.0	112.6	76.6	53.2	6.6	3.6	86.0	59.3	56.0	19.7
9	M	TOF	436	743	1.83	1.90	40.1	49.3	211.6	126.9	126.7	64.4	5.0	4.5	84.9	62.6	48.0	2.2
Mean			120	197	1.52	1.53	46.3	47.5	169.8	125.9	91.4	66.8	5.5	4.0	80.2	58.1	46.1	13.7
SD					0.19	0.21	5.60	3.85	21.18	15.28	16.06	7.92	0.87	0.60	9.33	9.00	5.99	7.54
P					0.472		0.553		0.008		0.011		0.015		0.008		0.008	

Abbreviations: BSA, body surface area; Dx, diagnosis; EF, ejection fraction; PA IVS, pulmonary atresia with intact ventricular septum; PR%, pulmonary regurgitation fraction; RVCI, right ventricular cardiac index; RVEDVI, right ventricular end-diastolic volume index; RVEF, right ventricular ejection fraction; RVESVI, right ventricular end-systolic volume index; RVSVI, right ventricular stroke volume index; SD, standard deviation; TOF, Tetralogy of Fallot.

Table S2. Forward and backward blood flow, cross-sectional area, and pulmonary regurgitation fraction in each pulmonary artery location before and after Pulsta valve implantation

**No**	**MPA**								**RPA**								**LPA**							
	**Forward Q** [ml/s]		**Backward Q** [ml/s]		**Area** [cm^2]		**PR%** [%]		**Forward Q** [ml/s]		**Backward Q** [ml/s]		**Area** [cm^2]		**PR%** [%]		**Forward Q** [ml/s]		**Backward Q** [ml/s]		**Area** [cm^2]		**PR%** [%]	
	pre	post	pre	post	pre	post	pre	post	pre	post	pre	post	pre	Post	Pre	post	pre	post	pre	post	pre	post	pre	post
1	118.5	70.3	52.3	8.2	4.48	3.42	44.1	11.7	63.9	46.9	24.3	6.3	2.88	2.40	38.0	13.3	55.0	23.2	27.8	1.9	1.33	3.25	50.5	8.3
2	90.9	72.2	48.4	11.2	5.11	6.28	53.3	15.4	54.5	41.7	30.0	6.6	1.93	3.36	55.0	15.8	36.6	30.5	18.2	4.6	1.81	1.68	49.7	15.0
3	98.1	73.5	53.5	2.7	5.29	5.54	54.6	3.6	67.0	33.8	38.4	1.1	2.18	3.11	57.3	3.4	30.9	39.9	15.0	1.6	3.62	4.43	48.7	3.9
4	128.8	82.8	48.2	18.6	8.04	7.00	37.4	22.4	75.8	47.8	27.6	10.7	2.53	3.84	36.4	22.3	52.8	35.3	20.2	7.8	2.73	4.24	38.2	22.0
5	102.9	27.8	52.6	5.8	5.91	4.64	51.1	20.7	47.9	5.6	22.6	1.1	1.15	2.65	47.2	19.4	54.7	22.4	30.9	4.7	2.18	3.08	56.5	21.2
6	101.3	93.9	38.7	20.2	5.09	5.07	38.2	21.5	69.2	47.7	26.2	10.7	1.54	2.33	37.9	22.5	35.2	46.4	15.7	9.4	2.28	1.04	44.7	20.3
7	142.7	78.8	61.4	13.9	4.66	3.72	43.0	17.6	80.3	43.3	32.0	7.5	2.32	1.87	39.8	17.3	62.9	34.4	29.4	6.0	4.21	2.33	46.7	17.6
8	110.3	49.5	59.8	14.2	5.40	6.07	54.2	28.7	56.8	27.9	31.2	8.3	1.74	1.96	54.9	29.9	53.3	21.8	28.9	5.8	3.03	3.47	54.3	26.5
9	180.0	118.2	84.0	1.4	4.98	7.14	46.7	1.2	146.1	103.1	70.3	1.2	2.86	3.42	48.1	1.2	34.6	18.1	14.1	0.1	1.54	2.86	40.6	0.7
Mean	119.3	74.1	55.4	10.7	5.44	5.43	46.9	15.8	73.5	44.2	33.6	5.9	2.13	2.77	46.1	16.1	46.2	30.2	22.2	4.7	2.53	2.93	47.8	15.1
SD	28.0	25.5	12.6	6.7	1.1	1.3	6.7	9.0	29.1	25.9	14.5	3.9	0.6	0.7	8.3	9.2	11.7	9.5	6.9	3.0	1.0	1.1	5.9	8.9
p	0.008		0.008		0.859		0.008		0.008		0.008		0.038		0.008		0.038		0.008		0.314		0.008	

Abbreviations: LPA, left pulmonary artery; MPA, main pulmonary artery; PR%, pulmonary regurgitation fraction; Q, blood flow rate; Re, Reynolds number; RPA, right pulmonary artery; SD, standard deviation.

Table S3. Correlations of vorticity in the pulmonary artery locations with non-CFD factors and other CFD measurements.

		With MPA vorticity				With RPA vorticity				With LPA vorticity			
		Univariable		Multivariable		Univariable		Multivariable		Univariable		Multivariable	
		Coefficient B	P	Coefficient B	P	Coefficient B	P	Coefficient B	P	Coefficient B	P	Coefficient B	P
Clinical information	shunt op	22.921±25.206	0.118			73.639±195.143	0.484			113.703±146.595	0.172		
	age at op	-0.686±2.118	0.545			1.175±14.494	0.878			-4.302±11.689	0.494		
	age at PPVI	-4.61±12.65	0.642			-15.573±25.381	0.268			-15.609±20.186	0.173		
EchoCG	PS peak velocity	-0.758±38.719	0.97			212.486±204.627	0.081			128.057±193.404	0.235		
	TR grade	14.146±28.528	0.363			-89.099±191.572	0.392			-14.439±168.733	0.872		
	TAPSE	2.25±4.622	0.372			-20.756±28.927	0.202			-12.848±25.624	0.358		
	LVEF	0.469±2.275	0.698			-8.585±13.963	0.267			0.724±12.791	0.915		
CT	MPA shape	-10.452±29.389	0.508			87.731±191.919	0.400			30.455±167.56	0.732		
	RVOT aneurysm	-6.23±31.702	0.712			39.573±211.564	0.725			70.07±170.49	0.447		
	Branch PAS	23.674±31.812	0.188			213.891±183.154	0.056			109.069±185.223	0.286		
Cath	RVSP	0.552±2.705	0.703			6.136±16.721	0.499			4.315±13.845	0.564		
	RVEDP	-1.023±5.995	0.748			2.395±40.245	0.910			-11.85±32.447	0.497		
	Elevated RVEDP	2.085±31.999	0.902			-64.262±208.212	0.564			-162.115±131.675	**0.0466**		
	Peak PG	2.818±3.865	0.203			2.677±28.372	0.859			-1.824±23.25	0.883		
	PVR	-4.091±17.57	0.662			90.183±98.317	0.115			50.815±91.763	0.314		
MRI	RVEF	0.16±2.856	0.916			-2.32±18.979	0.818			4.013±15.621	0.63		
	RVEDVI	0.872±0.393	**0.00336**			-0.183±5.041	0.945			0.816±4.164	0.712		
	RVESVI	0.833±0.784	0.0756			0.356±6.645	0.919			-0.221±5.547	0.94		
	RVSVI	1.661±1.197	**0.0297**			1.152±11.414	0.849			5.895±8.494	0.216		
CFD	MPA forward vel.	2.749±1.09	**0.00167**	2.749±1.09	**0.00167**	-3.599±15.166	0.656			-2.033±12.76	0.764		
	RPA forward vel.	1.011±1.596	0.255			10.26±8.964	0.060			2.282±9.66	0.657		
	LPA forward vel.	0.869±1.629	0.33			-3.847±11.323	0.527			6.429±8.499	0.182		
	MPA backward vel.	5.639±2.76	**0.00516**			-10.371±32.484	0.551			-8.386±27.151	0.564		
	RPA backward vel.	1.498±3.165	0.384			13.865±19.859	0.213			-1.564±18.62	0.874		
	LPA backward vel.	1.267±3.094	0.449			-5.756±21.132	0.610			12.922±15.229	0.14		
	MPA Vo	Na	Na			0.52±4.924	0.842			0.833±4.075	0.701		
	RPA Vo	0.012±0.111	0.842			Na	Na			0.56±0.459	**0.048**		
	LPA Vo	0.027±0.132	0.701			0.804±0.659	**0.048**			Na	Na		
	ED	0.024±0.236	0.849			1.871±0.751	**0.002**	1.871±0.751	**0.002**	1.135±1.012	0.064	0.995±0.849	**0.03**
	MPA area / BSA	-29.201±21.664	**0.0333**			27.52±203.089	0.798			20.06±169.662	0.823		
	RPA area / BSA	16.162±37.594	0.427			-214±209.9	0.086			-25.485±218.671	0.826		
	LPA area / BSA	-10.429±23.183	0.407			-14.797±162.55	0.863			-82.086±121.582	0.227		

Abbreviations: BSA, body surface area; BPA, branch pulmonary artery; ED, energy dissipation; LPA, left pulmonary artery; LVEF, left ventricular ejection fraction; MPA, main pulmonary artery; op, operation; PG, pressure gradient; PPVI, percutaneous pulmonary valve implantation; PS, pulmonary stenosis; RPA,

right pulmonary artery; RVEDP, right ventricular end diastolic pressure; RVEDVI, right ventricular end diastolic volume index; RVESVI, right ventricular end systolic volume index; RVOT, right ventricular outflow tract; RVSP, right ventricular systolic pressure; RVSVI, right ventricular stroke volume index; vel., velocity; TAPSE, tricuspid annular plane systolic excursion; TR, tricuspid regurgitation; Vo, vorticity;
